# Using Twitter to collect a multi-dialectal corpus of Albanian using advanced geotagging and dialect modeling

**DOI:** 10.1371/journal.pone.0294284

**Published:** 2023-11-27

**Authors:** Ercan Canhasi, Rexhep Shijaku

**Affiliations:** Faculty of Computer Science, University of Prizren, Prizren, Kosova; National University of Science and Technology, PAKISTAN

## Abstract

In this study, we present the acquisition and categorization of a geographically-informed, multi-dialectal Albanian National Corpus, derived from Twitter data. The primary dialects from three distinct regions—Albania, Kosovo, and North Macedonia—are considered. The assembled publicly available dataset encompasses anonymized user information, user-generated tweets, auxiliary tweet-related data, and annotations corresponding to dialect categories. Utilizing a highly automated scraping approach, we initially identified over 1,000 Twitter users with discernible locations who actively employ at least one of the targeted Albanian dialects. Subsequent data extraction phases yielded an augmentation of the preliminary dataset with an additional 1,500 Twitterers. The study also explores the application of advanced geotagging techniques to expedite corpus generation. Alongside experimentation with diverse classification methodologies, comprehensive feature engineering and feature selection investigations were conducted. A subjective assessment is conducted using human annotators, which demonstrates that humans achieve significantly lower accuracy rates in comparison to machine learning (ML) models. Our findings indicate that machine learning algorithms are proficient in accurately differentiating various Albanian dialects, even when analyzing individual tweets. A meticulous evaluation of the most salient attributes of top-performing algorithms provides insights into the decision-making mechanisms utilized by these models. Remarkably, our investigation revealed numerous dialectal patterns that, despite being familiar to human annotators, have not been widely acknowledged within the broader scientific community.

## 1 Introduction

Dialects constitute a critical facet of human linguistic and communicative behavior and are delineated as linguistic variations that diverge in phonology, grammar, and lexicon. Predominantly observed in geographically distinct regions, dialects embody cultural, social, and historical influences that have molded regional speech and language utilization. Despite their distinctive attributes, dialects remain components of comprehensive language systems and generally exhibit mutual intelligibility with other varieties of the same language [1].

Dialect identification, a task within the domain of natural language processing, entails the automated classification of text according to its dialect. This intricate task bears significance for numerous applications, such as machine translation and sentiment analysis. Dialect identification presents increased complexity when required to categorize exceptionally brief texts, including social media posts or tweets. Owing to the conciseness of these texts, limited linguistic information may be accessible for accurate classification. Consequently, productive dialect identification often necessitates the employment of sophisticated machine-learning algorithms and high-caliber training data.

**Fig 1:**
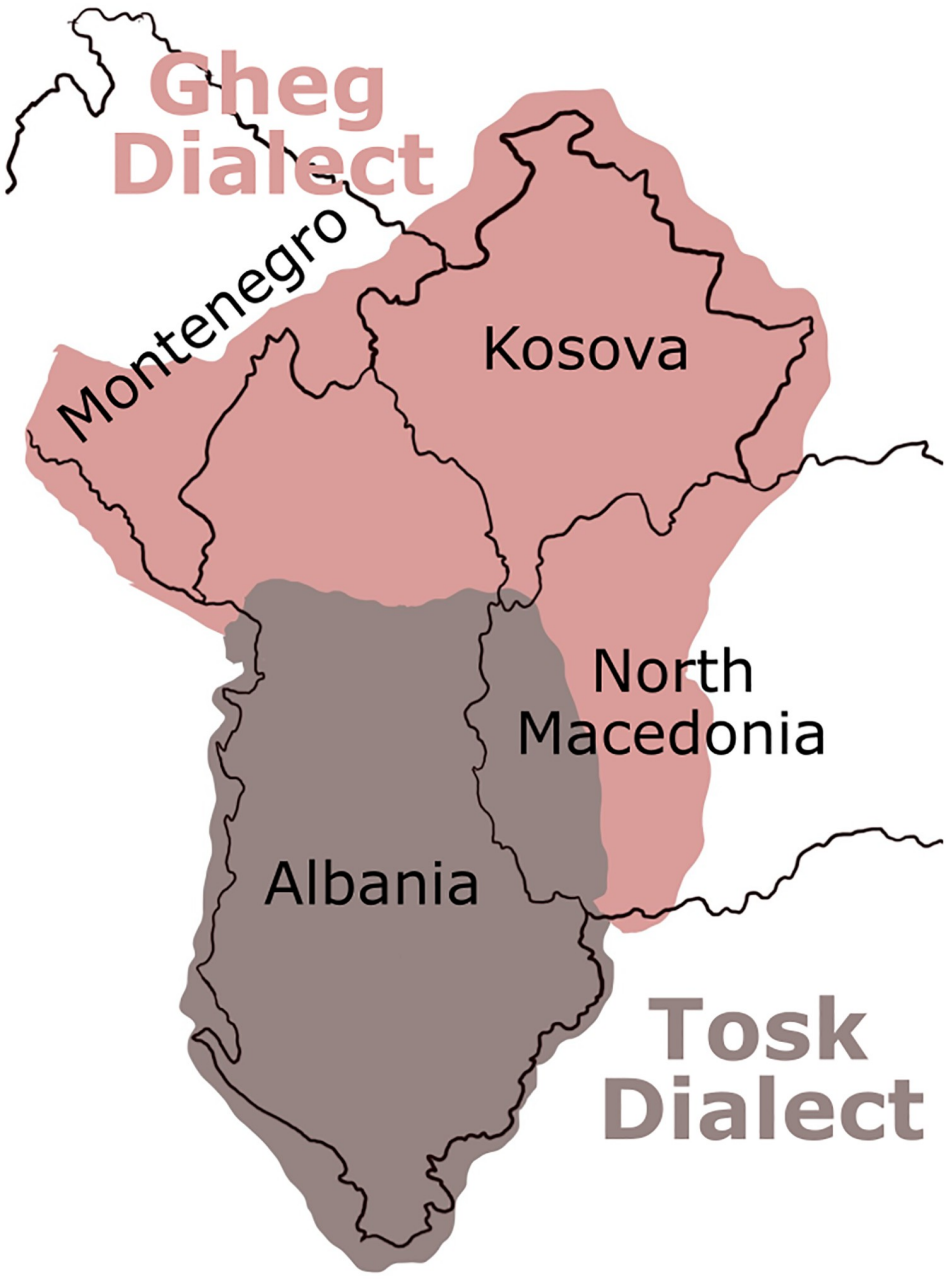
Different Albanian dialects in western Balkans.

In the present study, we confront the challenge of distinguishing dialects in tweets, imposing the constraint that we recognize language not solely at the tweet level but also at the level of the individual Twitter user. The three dialects under examination are spoken in three distinct regions, specifically Albania (AL), Kosovo (KS), and North Macedonia (MK). Considerable geographical distinctions exist among Albanian dialects within countries, across national boundaries, and even between urban and rural areas, as illustrated in Fig 1 The dialects studied in this paper belong to the Albanian language, an Indo-European language spoken by Albanians in the Balkans, as well as the Albanian diaspora residing in the Americas, Europe, and Oceania.

The Albanian language encompasses various linguistic varieties, including a standard form known as Standard Albanian (SA), which is utilized in official communications, newspapers, and other formal contexts. Additionally, numerous regional dialects exist in both spoken and written forms [2–4]. A successful attempt to compile the Albanian corpus, the first of its kind by size and quality, is described in [5]. These dialects are traditionally bifurcated into two primary groups: the northern Gheg dialects and the southern Tosk dialects. The demarcation line between these dialects is customarily identified as the Shkumbin River, which traverses central Albania along the east-west axis at approximately the 41st parallel north.

In Kosovo and North Macedonia, the predominant dialects are of the Gheg variety, whereas the dialects spoken in northwestern Greece belong to the Tosk category. Despite being classified as Tosk dialects, Arvanitika (historically spoken in Attica and Boeotia, Greece) and Arbëresh (spoken in southern Italy and Sicily) are frequently regarded as significant Albanian dialects. The emergence of these dialects in their respective regions can be traced back to the late 15th century, following the Ottoman conquest of the western Balkans. The linguistic traditions of these dialects have been preserved to the present day.

In digital communication, both Standard Albanian and its various dialects are frequently employed and are often intermingled within the same context. As anticipated, all dialects utilize a consistent phonemic inventory and adhere to the orthographic principle of writing as one speaks. Substantial overlap exists in the lexicon shared by these dialects, with some words differing by merely a single phoneme due to phonological, morphological, and etymological factors.

While minor grammatical discrepancies in phonology, morphology, and syntax can be observed among the dialects, these variations are relatively sparse and exert minimal impact on mutual intelligibility.

A pertinent inquiry in this scenario is whether it is possible to develop a machine learning model capable of accurately differentiating text samples authored by individuals in three distinct countries, each speaking a unique Albanian dialect. Should such a model be successfully trained, the question arises as to what the distinguishing characteristics utilized by the model are.

The multi-dialectal Albanian Text Corpus, encompassing text samples procured from Twitter, enables us to address pertinent research inquiries. Furthermore, our corpus functions as a crucial benchmark for evaluating the efficacy of dialect identification techniques. To achieve this objective, we investigate numerous traditional machine learning training methodologies and conduct experiments utilizing a deep learning approach.

Existing Research Problems:

- Sparse High-Quality Datasets: Existing computational linguistics studies often suffer from a lack of high-quality, geographically tagged datasets representing multiple dialects, especially for lesser-studied languages like Albanian.
- Complexity in Brief Texts: Tweets and other short social media posts provide limited context, making dialect identification a particularly challenging problem.
- Geographical Attribution: Current methods often struggle with accurate geolocation of social media users who do not provide explicit location data, a crucial factor in dialect identification.

The primary contributions of this paper include:

- The development, compilation, and public release of a novel Albanian Corpus of Twitterer-level annotated texts extracted from contemporary social networks. This reference corpus is specifically designed for dialect identification tasks, and we posit that it can contribute to numerous analogous, yet limited, investigations in the domain of natural language processing for the Albanian language.
- A demonstration of the advanced geotagging techniques used to facilitate corpus creation. The problem of determining the geographic location of a user who has not provided an explicit location is framed as a classification task, in which the country with the highest probability is selected from a set of known locations associated with the user’s social connections. To accomplish this task, we utilize the XGboost classifier in conjunction with multiple features that capture various attributes and properties of the Twitter user network.
- The training and evaluation of various Albanian dialect classification models, both quantitatively and qualitatively, employing our novel corpus.
- To validate the core assertions of this study, a comprehensive series of experiments is included that have been meticulously designed and executed–providing empirical evidence to support the underlying hypotheses and research questions.

The remainder of this paper is organized as follows. Related work is discussed in Section 2. In Section 3, we present the proposed novel Albanian dialect corpus compilation. In Section 4, we describe the experiments conducted, while in Section 5, the results of the paper are provided. Section 6. gives the conclusion.

## 2 Related work

### 2.1 Dialect identification

The automatic identification of dialects has evolved considerably over the years, transitioning from rule-based and statistical methods to advanced machine learning algorithms. Despite the advancements, there are key deficiencies that persist across various approaches.

#### 2.1.1 Rule-based and statistical methods

Early works such as Mustonen [6] focused on rule-based algorithms, highlighting lexical, morphological, and syntactic markers that are dialect-specific. For example, Padró and Padró [7] reported an impressive 99% accuracy in distinguishing between Spanish and Catalan using second-order character-level Markov models. While effective for well-defined dialects, these rule-based methods suffer from scalability issues, especially for languages with numerous dialects or for those that exhibit a high degree of lexical overlap.

#### 2.1.2 Machine learning approaches

Machine learning methods, including both supervised and unsupervised learning, have become increasingly popular. Ranaivo-Malancon [8] utilized character trigrams, exclusive words, and number formats in a semi-supervised model for Indonesian and Malay. Although these methods offer better scalability than rule-based approaches, they are often reliant on large labeled datasets, which are not always available for low-resource languages.

#### 2.1.3 Deep learning innovations

The rise of deep learning techniques such as convolutional neural networks (CNN) [9] and recurrent neural networks (RNN) [9, 10] has been a game-changer. These approaches can capture intricate patterns that simpler methods overlook. Nevertheless, they come with their own set of limitations, including computational cost and interpretability. For instance, while transformer-based models [11] have shown tremendous promise, their complexity and resource requirements make them impractical for real-time applications or use on low-end devices.

### 2.2 Dialect identification for low-resource languages and corpora

This subsection focuses on related works from the aspect of low-resource languages.

Padró and Padró [7] used second-order character-level Markov models to differentiate between Spanish and Catalan, achieving up to 99% accuracy. Ranaivo-Malancon [8] developed a semi-supervised model for distinguishing Indonesian and Malay, utilizing character trigrams, exclusive words, and number formats. Huang and Lee [12] employed a bag-of-words approach to classifying Chinese texts from mainland China and Taiwan with up to 92% accuracy. Moreover, Zampieri and Gebre [13] used a log-likelihood estimation method with Laplace smoothing to identify Brazilian and European Portuguese, obtaining 99.5% accuracy.

Ljubešić et al. [14] showed that Croatian and Serbian could be differentiated with 99% accuracy using a second-order character Markov chain and a list of forbidden words. Tiedemann and Ljubešić [15] added Bosnian to the language list, reaching 97% accuracy by identifying blacklisted words. Similarly, Ljubešić et al. [16] attempted to discriminate between Croatian and Serbian on Twitter data, using word unigram language models built from web corpora. The analysis revealed substantial Twitter activity from Bosnian and Montenegrin speakers, suggesting a 4-class schema considering all languages in the collection.

The Nordic Dialect Corpus [17] encompasses approximately 466,000 spoken words originating from Denmark, Faroe Islands, Iceland, Norway, and Sweden, with each dialect transcribed according to the standard official orthography of the respective country. In contrast, the ACTIV-ES corpus [18] represents informal language usage by Spanish speakers from Argentina, Mexico, and Spain and consists of 430 television or movie subtitle files. The DSL corpus collection [19] assembles news data from diverse corpora to simulate heterogeneous news content in various languages, incorporating six language variety groups and containing 18,000 training sentences, 2,000 validation sentences, and 1,000 test sentences for each language.

The ArchiMob corpus [20] comprises manually annotated Swiss German speech transcripts, gathered from four distinct regions: Basel, Bern, Lucerne, and Zurich, and was employed in the 2017 and 2018 VarDial Evaluation Campaigns. Furthermore, Kumar et al. [21] developed a corpus encompassing five Indian dialects with a total of 307,000 sentences obtained through scanning, processing with an OCR engine, and proofreading printed stories, novels, and essays sourced from books, magazines, or newspapers. Butnaru and Ionescu in [22] presented MOROCO, a dataset of 33,564 online news reports collected from the Republic of Moldova and Romania.

### 2.3 Twitter as a source for dialectal

In recent years, social media platforms such as Twitter have become a valuable source for collecting dialectal data due to their wide adoption and the diverse linguistic content generated by their Twitterers [23]. Researchers have utilized Twitter data to study various aspects of dialectology, such as lexical variation [24], phonological differences, and syntactic variation [25]. Additionally, Twitter data has been used to create multi-dialectal corpora for several languages, including Arabic [26] and English [27].

Geotagging, a technique that associates textual content with geographical locations, has played a crucial role in the development of these corpora, enabling researchers to assign dialect labels based on the geographic origins of the Twitterers [28].

The study [29] critically analyzes the geolocation prediction in Twitter using social networks. The authors discuss methods of predicting Twitterer locations, examine the role of network features in geolocation, and suggest that the follower-followee relationship may be an important factor.

Although the study in [30] mainly focuses on predicting social unrest, it touches on the relationships between local Twitterers and influential ones. It explores how geolocation information can be extracted from unprocessed Twitter data, and how this information can be used for various analyses, including identifying local followers.

The authors in [31] compare data from Twitter’s Streaming API with Firehose, highlighting potential biases in the Streaming API data that could affect research results. In [32], the authors investigate the dynamics of the location field in user profiles on Twitter, illustrating how Twitterers provide location information and how this information changes over time.

A global mapping of Twitter usage discussing the geography of Twitter and the distribution of geolocated tweets is presented in [33]. Graham et al. (2014) [34] analyzed the geolocation and language identification of tweets, showing the spatial and linguistic distribution of Twitterers worldwide.

## 3 The AlbDialectCorpus

The experimental dataset utilized in this study comprises tweets from 2,503 randomly selected Twitter users, which were gathered using the proprietary tools detailed in this chapter. Table 1 provides additional statistics regarding the novel dialect corpus. A limited pool of four annotators was available for the annotation process. The inclusion of other languages in the dataset was not permitted. Secondary languages, such as English, Bosnian, Italian, and Turkish, are commonly employed in the selected regions. For example, Twitter users from the Kosovo region, in addition to employing Albanian language (both standard and local dialects) and English, frequently use Turkish and Serbian/Bosnian/Croatian languages in their tweets.

**Table 1:** The textual statistics for DialectCorpus.

Regional Dialect	MK	KS	AL	Grand Total
**Number of Users**	835	867	801	2,503
**Avg. # Tweets per User**	61.67	55.07	87.01	68.25
**Avg. # Tokens per tweet**	9.94	11.13	17.28	12.78
**Total # of Tweets**	51,495	47,751	69,688	168,934
**Total # of Tokens**	512,279	531,810	1,204,220	2,248,309

**Fig 2:**
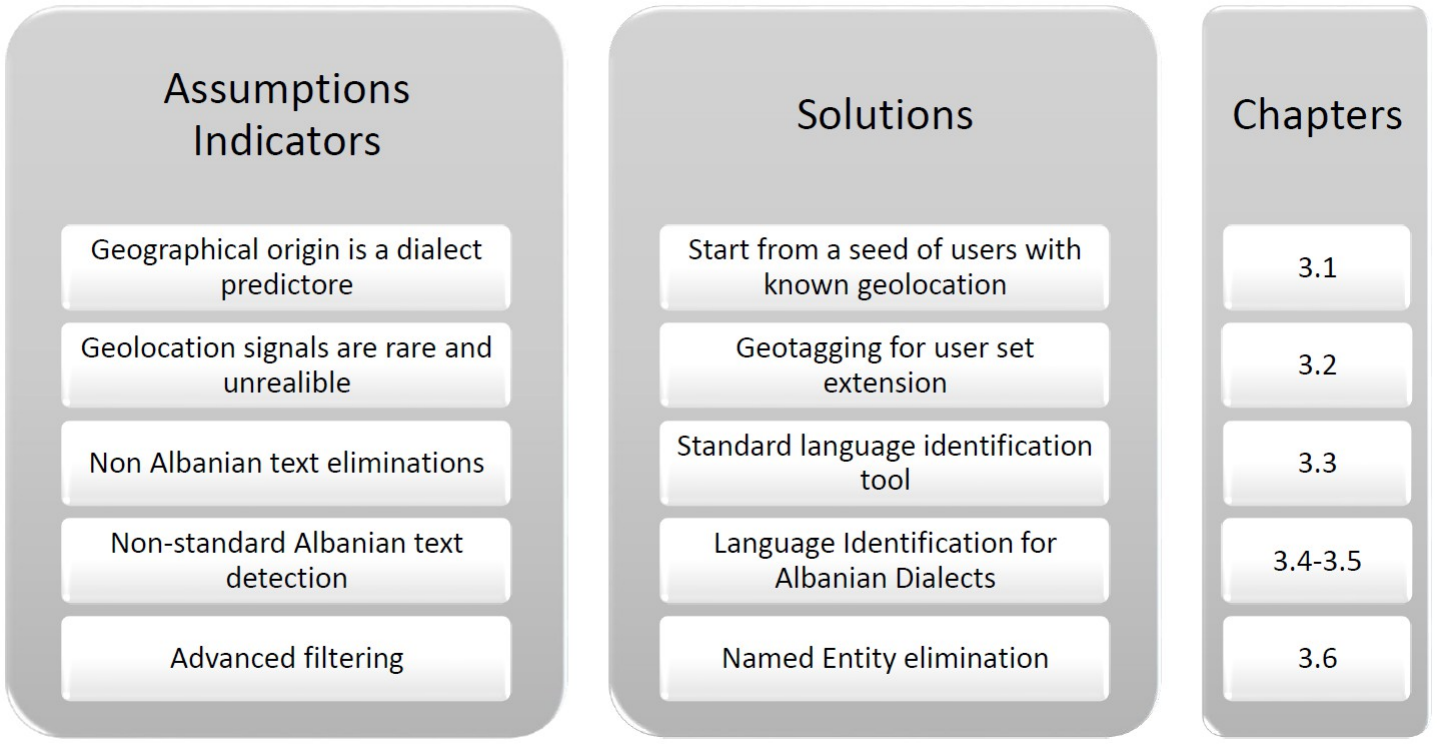
List of assumptions, proposed solutions, and related sub-chapters.

This chapter proceeds to explicate the methodology employed in assembling the corpus. To assist readers in comprehending the concepts presented in the remainder of the chapter, Fig 2 offers a visual representation of the ideas discussed.

It is important to highlight that The Albanian Dialect Corpus is readily accessible to the public and can be found on Zenodo at 10.5281/zenodo.8349099 or on GitHub at github.com/rexshijaku/albanian-dialect-corpus

### 3.1 Data gathering

Our goal in data gathering was to compile a corpus that includes texts written and shared in different Albanian dialects by Twitterers of three different regions where the Albanian language is spoken.

For this study, we chose to use Twitter as our source of data because it is one of the most popular social networks, with around 450 million active Twitterers. Additionally, Twitter has a publicly accessible API [35], making it easy to collect data from the platform. Twitter is also an interest-graph network, which means that Twitterers can form connections with others based on shared interests, regardless of whether they know each other in real life. This means that online relationships on Twitter are not always dependent on geography, which can introduce noise and complexity into the social network graph. Furthermore, Twitter allows for unidirectional social connections, where a user can follow another user without the need for reciprocation. These types of relationships can create ties that make it challenging to perform network-based geolocation of Twitterers. Overall, the combination of these factors makes Twitter a valuable source of data for studying dialect variation in Albanian.

One of the key challenges in constructing a corpus of Albanian dialect texts is the need to make a series of assumptions about the language and its variations. In this section, we explain how we approach this challenge, and how we use our assumptions to iteratively create the corpus. By carefully selecting and combining a series of assumptions about Albanian dialects, we can iteratively construct a novel corpus of texts. Our approach is designed to capture the rich diversity and complexity of Albanian dialects, while also addressing the unique challenges and opportunities that arise in this process.

Our assumption that the geographical origin of a user is a strong predictor of the dialect they use in their writing is a crucial part of our approach to building the corpus [34, 36, 37]. The relationship between language and geography has been a topic of interest to linguists since the nineteenth century [36].

While it is possible for a user to write in multiple languages and dialects, we used advanced filtering techniques to isolate only the tweets written in the Albanian dialect used in a particular region. This assumption guides the first step in our corpus compilation process, which involves identifying Twitterers from the geolocations we are interested in. By carefully selecting and combining a series of assumptions about Albanian dialects, we can iteratively construct a novel and comprehensive corpus of texts. Our approach is designed to capture the rich diversity of Albanian dialects, and to provide a valuable resource for researchers and language enthusiasts alike.

According to Twitter documentation, location-related data for a given tweet is provided in two ways: (1) tweet location, representing the locational metadata associated with the tweet itself, and (2) account location representing the ‘home’ location provided by the Twitterer’s profile. It is important to notice that the latter is a free-form character field, and may or may not contain metadata that can be geo-referenced. Twitter reports that only 1-2% of tweets shared have an associated location [31–34].

We observe the same behavior pattern in Albanian Twitter usage, namely the very low percentage of Twitterers explicitly reporting their tweets region. To tackle this challenge, we created our second assumption and designed a specific method to treat the given problem.

Our third assumption is that the followers of a locally famous politician, journalist, or influencer tend to be Twitterers from that same area. This assumption is based on the idea that famous people, as measured by the number of followers they have on Twitter, are likely to have a strong local presence and influence. Using this assumption, we can infer the region from which the Twitterer writes based on the Twitterers they follow or who follow them [28–30, 38, 39].

Our strategy which is substantially similar to the previous approaches [28–30, 38, 40] is based on iteratively (at least for two iterations) enriching the list of locatable Twitterers using following-follower relationships. Verification is achieved using cross-validation techniques, in which the location of a fraction of the Twitterers with known locations is used to determine the location of the others–thus allowing us to compare the actual location to the inferred one and verify the quality of the estimation. With an estimate of the precision of the method, it can then be applied to locations of tweets. We intend to infer the location of as many Twitterers as possible to increase the number of tweets that can be used in spatial analyses of the social phenomena.

**Fig 3:**
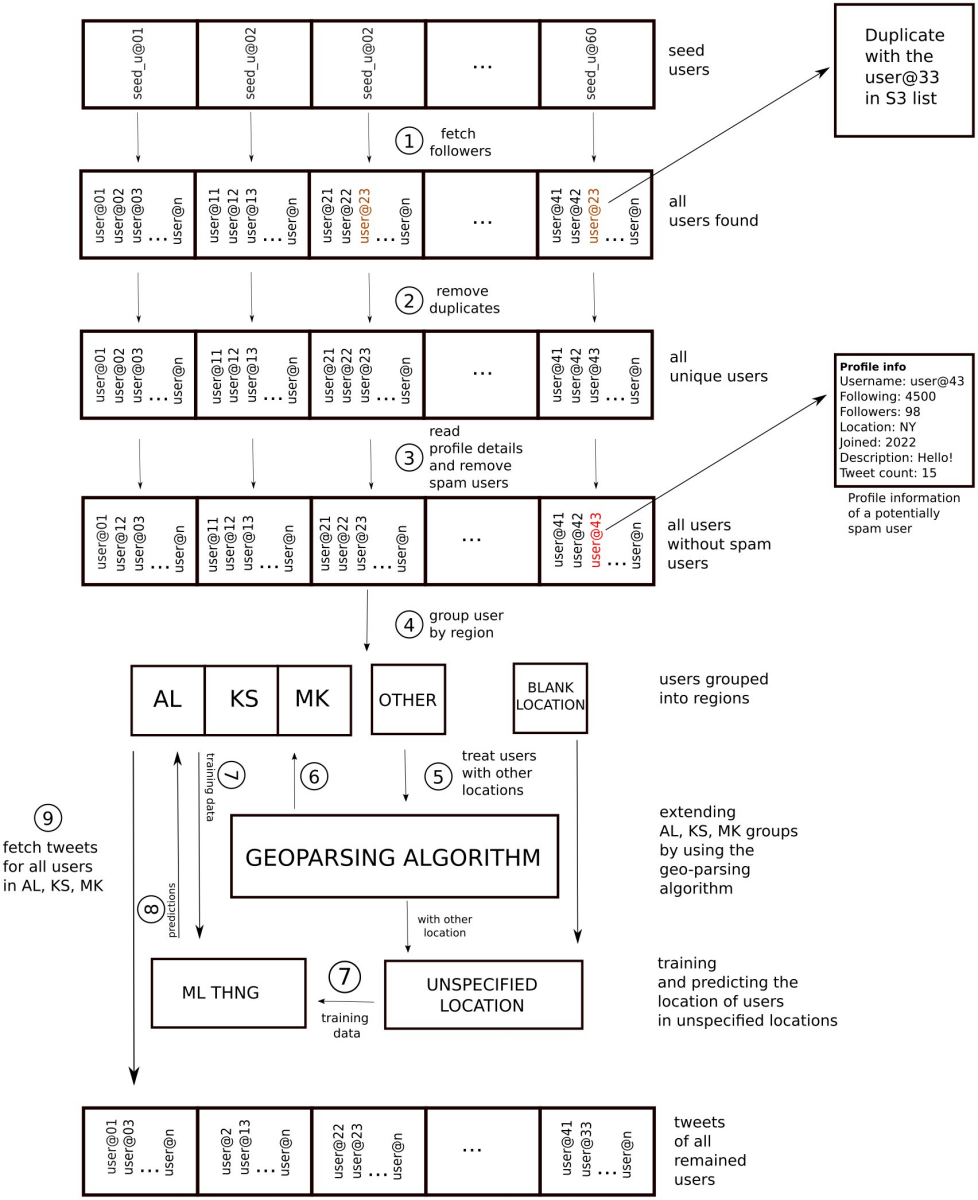
The visual description of the methodology used for using Twitter to collect a multi-dialectal corpus of Albanian.

By the proposed analytical approach, an initial seed of 60 Twitterers was manually defined, with 20 users representing each targeted region. A schematic representation of the methodology employed for the utilization of Twitter in the collection of a multi-dialectal Albanian corpus can be found in Fig 3. The initial list comprised politicians from diverse political affiliations, journalists, entrepreneurs, influencers, and ordinary Twitter users. These individuals were selected from geographically dispersed locations, varying popularity levels, and diverse backgrounds, to maximize tweet author heterogeneity and ensure appropriate randomization across target regions. We selected seed users from Kosovo and Albania, while for seed users of North Macedonia, we employed external assistance. An average of 430 followers were obtained for each identified Twitter user, resulting in a total of 25,939 users. These users were acquired from the follower list of each seed user in the default order provided by the Twitter API. At this stage, only the unique identifiers, or usernames, of each author were collected. To reduce computational efforts, duplicate users were eliminated, leaving 18,619 unique Twitter users.

Upon further examination, it was determined that the current list contained a substantial number of spam users [41], defined as users who follow more than two thousand other Twitter accounts. The threshold was established based on an outlier identification. After removing spam and invalid accounts, the final user dataset comprised 13,812 valid accounts. Despite employing a stringent collection mechanism, the gathered user dataset was further verified by examining explicit geotagging. A total of 6,220 users with accurate geotags were identified, including users specifying their location as ‘Kosovo’ (717), ‘Albania’ (768), and ‘North Macedonia’ (108). Users with implicit regional references or lacking relevant regional information were categorized as ‘other’. Due to the unrestricted nature of the location field, inconsistencies in location representation were observed. For instance, Prizren, a city in Kosovo, was referred to by its original name, shorthand (Pz), or car plate code (04). To address this issue, a geoparsing algorithm was developed to associate each text with a location, thereby significantly increasing the number of identified users in each region. Specifically, the algorithm doubled the user count for Kosovo and Albania and increased the North Macedonia user count by a factor of seven.

### 3.2 Twitter geolocation: The classification approach

We cast the problem of geotagging the remaining users lacking data as a machine-learning problem. More precisely, we treated the problem as a classification problem. The main rationale behind this decision was the existence of a set of 10,000 users with ground base truth, i.e. reliable location data. This set of users was used as training data. The process is known as geocoding, geoparsing, or geotagging, and can be used to manage the problem of missing location data in tweets [28, 29]

#### 3.2.1 Modeling

The presented model can be comprehensively characterized as a network-based geolocation approach, often referred to by the adage, “You are where your friends are.” This methodology seeks to employ the topological structure of a user’s social network to deduce their geographic location.

**Fig 4:**
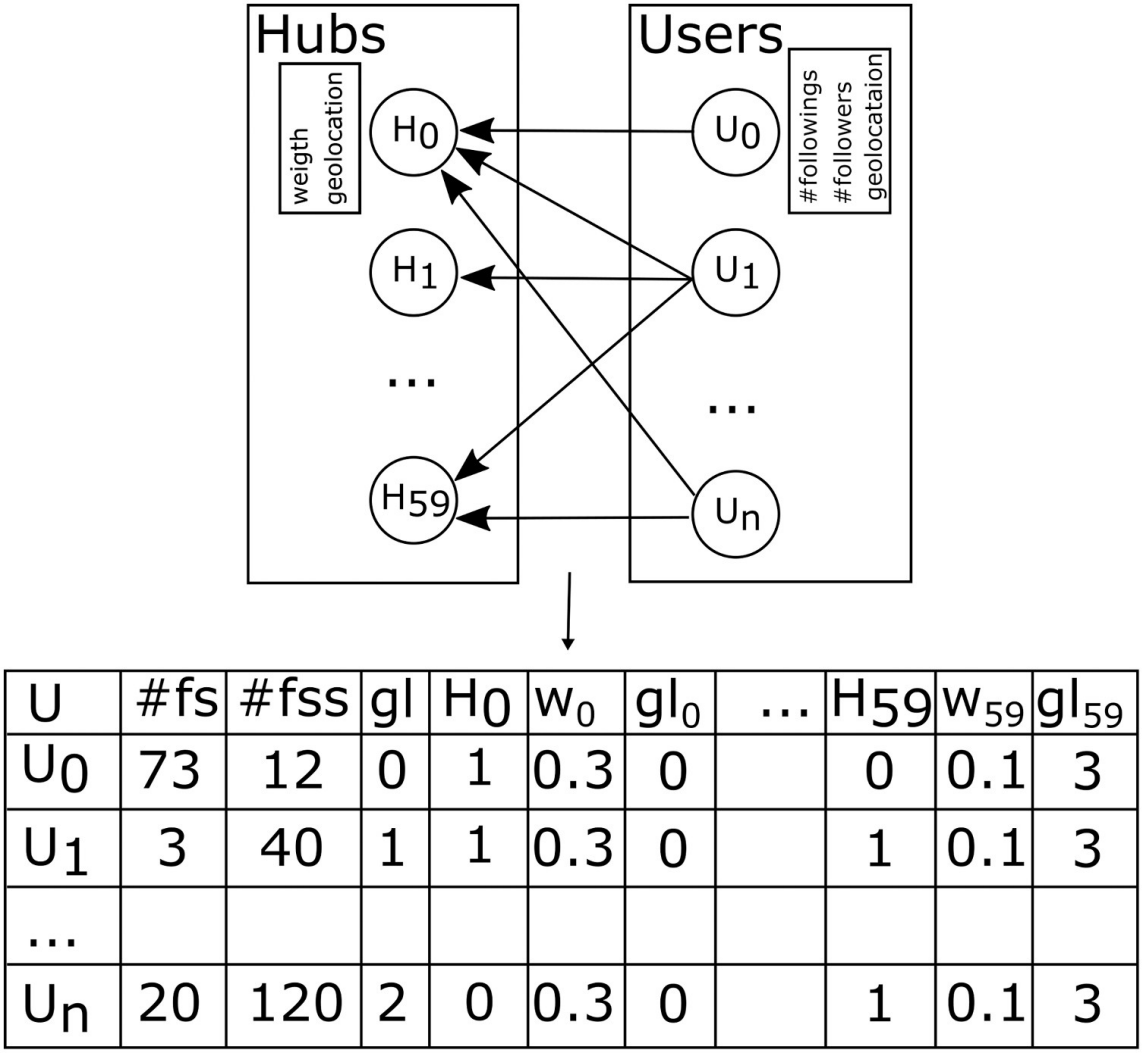
Displayed above is a bipartite-directed graph illustrating the relationships between standard Twitter users and hub Twitter users. Accompanying this graphical representation a tabular dataset is shown, which provides a structured depiction of the connections present within the aforementioned bipartite-directed graph.

In Fig 4, we visualize the graph representation of our problem modeling. In the same figure, the left side shows the hub users and the right side features the normal users. The arrows represent the binary user following hubs relations, while each hub and its users are additionally described by relevant properties such as relevant importance, geolocation, number of followers, and number of users following.

To convert the graph into ML-appropriate format, we convert the graph into a matrix shown in Fig 4.

By analyzing the spatial distribution and relationships among the user’s connections, the model endeavors to extrapolate the most probable location for the target user, leveraging the inherent spatial correlation within the network. This approach highlights the significance of social ties in providing valuable contextual information for geolocation estimation.

Table 2 showcases several dataset statistics. Because here we are dealing with the bipartite-directed graph, the median number of incoming/outgoing connections is almost equal to the average. The graph’s reciprocity is relatively low at 0%, indicating that a significant number of Twitter relationships are one-sided, meaning users do not always reciprocate the following.

**Table 2:** The summary of the network parameters.

Parameter	Value
Number of nodes	13870
Number of edges	59928
Average in-degree	4.321
Average out-degree	4.321
Number of users	13870
Mean in-degree	4.321
Mean out-degree	4.321
Reciprocity	0.0

#### 3.2.2 Feature engineering

For each data point in our user dataset, we extracted an extensive set of 180 features to facilitate a comprehensive analysis. The initial step in our data collection process involved the identification of 60 influential accounts, which will be referred to as the “hub list” throughout the remainder of this chapter. A substantial portion of the features is derived from the relationship between individual users and the hub list, primarily focusing on follower-following dynamics.

By examining the interconnections between users and the hub list, we can better understand the significance of these relationships within the context of the larger social network. The comprehensive set of features enables a more robust analysis, incorporating various dimensions of user behavior and interaction patterns, which ultimately contributes to a more accurate representation of the underlying social network structure.

The comprehensive set of derived features can be categorized into two distinct groups: structural features and social-network-hub-related data.

The first group encompasses three features extracted from the user’s Twitter profile, which include: (1) the number of users followed by the target user, (2) the number of followers of the target user, and (3) the age of the account in question.

The second group, comprising the remaining 180 features (three features for each of the 60 hubs), is related to the social-network-hub data. This group includes (1) binary user-to-hub following information, which indicates whether the target user is following a specific hub, (2) the weight assigned to each hub, reflecting its relative importance or influence within the network, and (3) a categorical value denoting one of the geolocations associated with the hub, providing spatial context to the analysis.

By integrating both structural and social-network-hub-related features, our analysis can capture a more comprehensive view of the user’s position within the social network, as well as their interactions with influential accounts. This rich feature set ultimately allows for a more accurate and nuanced understanding of the user’s behavior and geographic location.

The weight of a hub is calculated by the next formula:

$$\begin{array}{c} Weight(H_{i})=\alpha *Centrality\left(H_{i}\right)+\beta *F_{i}+\gamma *G_{i} \end{array}$$ (1)

In the formula the *Weight*(*H*_*i*_) represents the weight of *hub*_*i*_, *Centrality*(*H*_*i*_) denotes the centrality measure of *hub*_*i*_ within the network (e.g., degree centrality, betweenness centrality, etc.), *F*_*i*_ is the binary user-to-hub following information for *hub*_*i*_, (1 if the target user follows the hub, 0 otherwise), *G*_*i*_ is the categorical value representing one of the geolocations associated with *hub*_*i*_, *α*, *β*, and *γ* are coefficients that determine the relative importance of each component in the calculation (these values can be adjusted or optimized based on the specific application or analysis).

This formula incorporates the centrality measure of each hub, the binary user-to-hub following information, and the categorical geolocation value to calculate the overall weight of each hub in the network. The coefficients *α*, *β*, and *γ* allow for the fine-tuning of the relative contributions of each component to the final weight calculation.

#### 3.2.3 Experiments

To address the problem of predicting the location of a Twitter user based on structured and social-network-hub-related features, we propose a quantitative evaluation based on prediction. This experiment shows how accurately a classifier can distinguish between three different intended locations regarding the 183 features. We hypothesized empirically that the features could help to identify (predict) location without obtaining explicit location information. In this classification, a classifier assigns a ternary class (AL, KS, M) to each user.

In our classification formulation, there are *n* users *U* = *u*_1_, *u*_2_, …, *u*_*n*_. Each user in this formulation represents the users as a combination of 183 features. The classification task predicts whether *u*_*i*_ is AL, KS or MK utilizing a classifier *c* that assigns a label *l* to *u*_*i*_:

$$c:u_{i}\to \left\{l_{1},l_{2},l_{3}\right\}$$

The classifier uses a set of n features, *F* = *f*_1_, …, *f*_*n*_, obtained from the profile and tweets of users. To determine the class of each user, we utilized Random Forest. We used three-fold cross-validation, in which the data are broken into three subsets, and the holdout method is repeated three times. Each time, one of the three subsets is used as the test set and the other two subsets are used as the training set.

We experimented with various training algorithms and a subset of features but the model trained with XGBoost and a complete set of features offered the highest classification performance, including an accuracy of 87.40% and an F-measure of 0.852. The detailed results of the model evaluation are presented in Table 3.

**Table 3:** Classification report.

	precision	recall	f1-score	support
**KS**	0.81	0.90	0.85	423
**AL**	0.90	0.83	0.86	415
**MK**	0.79	0.72	0.75	155
**accuracy**		0.84		993
**macro avg**	0.83	0.82	0.82	993
**weighted avg**	0.84	0.84	0.84	993

Given the classification model, we inferred the location for 1.6K users with unknown locations. To further verify the results we manually checked users whose classification probability (score) was above 0.8.

### 3.3 Language filtering

Since our main goal was compiling the Albanian dialect identification corpus, the dataset prepared up to this point required the final processing to fulfill our original goal. Starting with the assumption that Tweets written by Albanian speakers from three different Albanian-speaking regions would mostly represent the three targeted Albanian dialects led us to the creation of a dataset containing a balanced number of users from those regions.

However, upon closer inspection of the tweets written by the selected users, it was found that some unwanted properties were present in the dataset. The most notable issue was that some local users wrote in more than one foreign language in addition to the targeted dialects. The most commonly used non-Albanian language in tweets was English, as expected. Other languages were typically regional, such as Serbian/Bosnian, Turkish, and German in Kosovo; Macedonian, Serbian, Bulgarian, and Turkish in Macedonia; and mostly Italian in Albania. This posed a challenge for the dialect identification model, as the presence of non-Albanian languages in the dataset could potentially interfere with the model’s ability to accurately identify the dialect of a given tweet. To address this issue, steps were taken to filter out tweets written in languages other than Albanian, resulting in a cleaner and more focused dataset for training the dialect identification model.

Consequently, we made a new assumption: “Given the high precision standard language identification tool, one can successfully eliminate non-Albanian language tweets”. Unfortunately, this assumption had a huge flaw. The standard language identification tools would identify Albanian dialect texts as non-Albanian texts since none of them are trained to classify the same as an Albanian sub-language class. As such, this approach would rule out the most valuable tweets.

Solving the new problem required a three-folded approach: 1) using a high-quality standard language identification tool, such as fastText [42]; 2) training a sub-optimal Albanian dialect identification model; and 3) algorithmic solution of the main problem based on 1) and 2).

#### 3.3.1 High-quality standard language identification tool

fastText [43] can recognize 176 languages. These models were trained on data from Wikipedia, Tatoeba, and SETimes, used under CC-BY-SA [42].

### 3.4 Suboptimal smoothed N-gram based models for tweet language identification for Albanian dialects

Standard language identification tools are unable to accurately recognize texts in Albanian dialects, as these tools have not been trained in such dialects. To address this limitation, we developed a rudimentary Albanian dialect identification model utilizing a subset of our initial tweet dataset. This subset was curated through the application of a straightforward selection criterion: inclusion of tweets containing at least one of the top 100 most frequent words from each of the three Albanian dialects under investigation.

#### 3.4.1 N-gram feature models

N-gram is a probabilistic language model widely used in tasks similar to ours. In computational linguistics, an n-gram is a contiguous sequence of ‘n’ items from a given sequence of text. These items can be phonemes, characters, words, and others. A language model attempts to reflect the frequency with which each item occurs as a sentence in a text.

#### 3.4.2 Smoothing techniques

To improve the estimated probabilities of the language models and increase the accuracy of the models as a whole, several smoothing techniques were proposed [44].

#### 3.4.3 Machine learning algorithm

The Log-Likelihood Ratio algorithm was employed in our experiments. As shown in Eq 3.4, *N* represents the number of *n*-grams in the test text, *L* denotes the language model, and *n*_*i*_ refers to the *i*-th *n*-gram. For a given test sample, the probability associated with each available language model is computed. Ultimately, the language model yielding the highest probability determines the language of the text [45, 46].

$$\begin{array}{c} P\left(L|\text{text}\right)=\underset{l}{\text{max}}\;(\sum_{i}(\text{log}\;P\left(n_{i}|L\right)+\text{log}\;P\left(L\right))) \end{array}$$

#### 3.4.4 Training protocol

**Fig 5:**
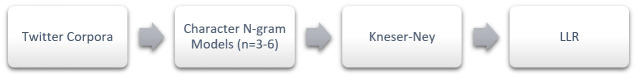
The overall language filtering workflow.

In our training protocol, we employed a log-likelihood ratio classification methodology founded on word n-gram language models, with n values ranging from 3 to 6. This technique entails estimating the probability of a specific text corresponding to each of the available language models, subsequently selecting the model that assigns the highest probability to the text as the predicted language. The general language filtering workflow is presented in Fig 5

Utilizing word n-grams (sequences comprising n words) as the fundamental units of the analysis enables capturing context and co-occurrence patterns of words in the text, which can offer valuable insights for language identification. By incorporating a spectrum of n values, our approach captures extensive contextual information, thereby enhancing the accuracy of the language identification model. Overall, this training protocol presents a robust and adaptable method for determining the language of a given text.

### 3.5 Language filtering algorithm

To generate a definitive list of tweets written solely in one of the Albanian dialects, we developed an algorithm following the procedure delineated in Listing 3.5. Initially, the algorithm employs a standard language identification tool to exclude tweets written in languages other than Albanian. Subsequently, it applies the Albanian dialect identification model—trained in the preceding step—to ascertain the dialect of each tweet in the remaining dataset. Ultimately, it selects tweets predicted to be in one of the three target Albanian dialects, yielding a final dataset comprising tweets exclusively in one of the Albanian dialects. This algorithm facilitates the creation of a precise and concentrated dataset for training the dialect identification model, thereby enhancing its performance and accuracy.

3.5 The Python code for language filtering algorithm

1 **def** get_dialect (tweets):

2  **for** each tweet **in** tweets:

3   lang, score = getTweetsLanguageProbaFromFasttext (tweet)

4   **if** score > 0.9 **and** lang == ‘sq’:

5    tweet [’dialect’] = ‘tosk_al’

6   **else if** score < 0.6:

7    dialect, scored = getTweetsDialectProbaITool (tweet)

8    **if** scored > 0.9:

9     tweet [’dialect’] = dialect

10  **return** tweets

The algorithm outlined in Listing 3.5 functions as follows: In line 3, it examines the original language of the tweet. If the language identification score is high and the language is identified as Albanian, the algorithm concludes that the tweet is composed in the standard Albanian Tosk dialect. If the original language is indeterminable (i.e., the language probability falls below 0.6), the tweet is presumed to be written in a non-standard language. Under such circumstances, the algorithm employs the dialect identification model to deduce the dialect of the tweet and the score assigned by the model. Utilizing this information, the algorithm discerns whether the tweet is composed in the Gheg dialect prevalent in Kosovo or the Macedonian Gheg dialect. This approach enables the algorithm to accurately classify the dialect of a given tweet, even when the tweet’s language deviates from standard Albanian. It is of crucial importance to remember that the algorithms and models presented in this section are merely used in compiling the corpus. The actual corpus evaluation and its utilization using machine learning methods are presented later in the paper.

### 3.6 Additional filtering steps

#### 3.6.1 Named entity removal

The initial experiment reveals that named entities can artificially raise the performance which is consistent with observations in previous works [22, 47]. Hence we decided to replace the named entities with a generic *ne* term. Since there aren’t any resources or tools for automatic NER, we used a rather simple, manually–crafted list for term replacements.

#### 3.6.2 Discriminative characteristics

To enhance comprehension of dialect classification performance, an examination of the most distinctive attributes is conducted. With the presence of named entities, the classifier selects–among other entities country–names (e.g., Kosovo, Albania, Macedonia), capital cities, and prominent political figures as distinguishing characteristics. In the absence of named entities, the classifier appears to opt for contextually relevant terminology for each class.

## 4 Experiments

This section provides an account of the experiments conducted and the resulting discussions. To address the research questions introduced in Section 1., we meticulously crafted a diverse range of experiments. Our experimental approach encompassed the use of:

1. Tweeter/text representation and selection
2. The set of traditional and some of the recent classification methods
3. Two main approaches to verify the quality of the dialect corpus: user classification and tweet classification
4. Advanced feature importance analysis and visualization using the Archetypal Analysis and SHAP values

We utilized the Albanian dialect corpus that we compiled to demonstrate its importance and practicality by implementing it in automatic dialect classification. To accomplish this task and determine the dialect of the content, we needed to create a text model for tweets. Therefore, in Section 4.1, we present the methodology used for text modeling. Subsequently, in Section 4.2, we provide a list of classification methods used in experiments, while in 4.3 the SHAP methodology is presented.

### 4.1 Text representation models

We represented each tweet/user’s text in four different ways that focused on the content of the text. These methods include the classic bag-of-words (BoW) approach, as well as three state-of-the-art distributed text representation techniques: Word2Vec [48], FastText [43], and BERT [49].

To score the weights of the tokens in each item, we used the TF-IDF (term frequency-inverse document frequency) method, which is also known as a BoW representation. The pre-trained word vectors were trained using Word2Vec, FastText, and BERT. Whenever possible, we used pre-trained models, but in some cases, we trained our models using a large set of Albanian language news documents that were crawled from the web over the last two years.

By using these four different methods, we were able to better understand the content of the tweet texts and make more accurate predictions about their veracity. This allowed us to improve the performance of our machine-learning models and better identify dialects of the Albanian language.

#### 4.1.1 Preprocessing

Before generating the feature vectors with BoW, Word2Vec, FastText, and BERT, all instances of our dataset were converted to lowercase. This is a common preprocessing step that ensures that words with the same spelling but different capitalization are treated as the same word. After converting the text to lowercase, we tokenized the documents based on whitespaces and punctuation marks. This step involves splitting the text into individual words, or tokens, which are then used to create the feature vectors.

### 4.2 Classification methods

The various dialect classification models were trained using a combination of classic methods and more recent techniques. The specific methods used in the training process included a variety of approaches that have been proven effective for dialect classification. Some of these methods included traditional machine learning algorithms, such as support vector machines and decision trees, as well as more modern techniques like deep learning. By using a diverse range of methods, we were able to train robust models that can accurately classify dialects in a variety of different contexts. The full list of methods is: 1. Logistic Regression (LR) [50]; 2. Naive Bayes [51]; 3. Support vector machines (SVM) [52]; 4. Decision trees (DT) [53]; 5. Random forest (RF) [54]; 6. KNN classification [55]; 7. XGBoost [56].

The well-known Python library Scikit-learn [57] includes implementations of many of the methods used to train the dialect classification models. Therefore, we primarily used this library for these methods, as well as for many other data science tasks such as feature selection and evaluation.

Since the goal of this work is not to optimize the parameters or hyperparameters of the methods, but rather to evaluate the quality of the proposed corpus classification results, we used the default values for these parameters. This allowed us to focus on assessing the overall performance of the models–rather than on fine-tuning their specific settings. By using the default values, we can more easily compare the results of the different methods and draw meaningful conclusions about their performance.

#### 4.2.1 Performance metrics

To compare the results of the dialect classification models, we employed several well-known performance measures for classification tasks. These measures allowed us to quantitatively evaluate the accuracy, precision, recall, and other key characteristics of the models. Some of the specific measures we used included the F1 score, which is a measure of the balance between precision and recall.

### 4.3 SHAP (SHapley Additive exPlanation) approach for results interpretation

Machine learning has the potential to be an incredibly useful tool in predicting dialects. However, many models do not explain their predictions, which can be a significant barrier to the adoption of machine learning. To address this issue, authors in [58] proposed a new approach called SHAP (Shapley Additive exPlanations). This approach is designed to help users interpret the predictions made by complex models, including LightGBM, NGBoost, CatBoost, XGBoost, and Scikit-learn tree models.

SHAP is based on game theory, specifically the work of Shapley in 1953. It allows users to understand the specific reasons why a model made a particular prediction for a given input (X) by calculating the impact of each feature on the prediction. This is done by estimating the Shapley value, which is calculated using a specific formula. By using SHAP, users can gain a better understanding of how their machine learning models are making predictions–and learn why certain features are more important than others. This can help to improve the accuracy and reliability of machine learning models, as well as increase their adoption and use.

$$\begin{array}{c} {\hat{\phi }}_{j}=\frac{1}{K}\sum_{k=1}^{K}((\hat{g}(x_{+j}^{m})-\hat{g}(x_{-j}^{m}))) \end{array}$$

where $\left(\hat{g}(x_{+j}^{m}\right)$ is the prediction for x, but with a random number of feature values.

In [59] TreeSHAP for gradient boosting models, among them XGBoost is proposed. TreeSHAP offers a rich visualization of each feature attribution that improves over classic feature importance and partial dependence plots.

## 5 Results

In this section, we present the results of our experiments on dialect classification using the newly collected multi-dialectal corpus of Albanian obtained from Twitter. Our goal is to explore the effectiveness of advanced geotagging and dialect modeling techniques in identifying the various dialects of Albanian present in the corpus. Identifying different Albanian dialects is challenging due to the considerable variation in phonology, morphology, and syntax across the dialects. Therefore, our approach involves the use of machine learning algorithms to learn patterns in the data and identify distinctive features that can be used to differentiate between the dialects.

### 5.1 The classification algorithm

We have conducted experiments with various classification algorithms to identify the best model or set of models. The aim is to use the best training algorithm for the remaining experiments. The summary of the classification results can be found in Table 4. The models were trained using the standard training parameters for each algorithm. For text modeling, we utilized TF-IDF on the word level, with an n-gram range of one to three and a maximum number of features set to 10,000. The input dataset included user- and tweet-level data points for the training algorithms.

**Table 4:** The F-1 score for each training algorithm, two dataset modes, and two TF-IDF modeling levels.

	User-level words	User-level chars	Tweet-level words	Tweet-level chars
MNB	0.906	0.872	0.817	0.804
DT	0.866	0.834	0.816	0.802
RF	0.905	0.836	0.817	0.804
XGBoost	0.898	0.868	0.431	0.802
KKN	0.888	0.774	0.507	0.738
LR	0.924	0.868	0.813	0.822
SVC	**0.941**	**0.934**	**0.819**	**0.821**
Fasttext	0.922	0.902	0.811	0.810

The bold values indicate the best scores.

Since the SVC and MNB algorithms reached the best F1-score on most of the modes of datasets, for the rest of the experiments we decided to use the SVC as a main training algorithm. As far as the text modeling is concerned, the best results are obtained when TF-IDF over n-grams of characters is used. Nevertheless, given the better interpretability of word-level TF-IDF modeling, we use this schema in the rest of the experiments.

### 5.2 The classification report

**Fig 6:**
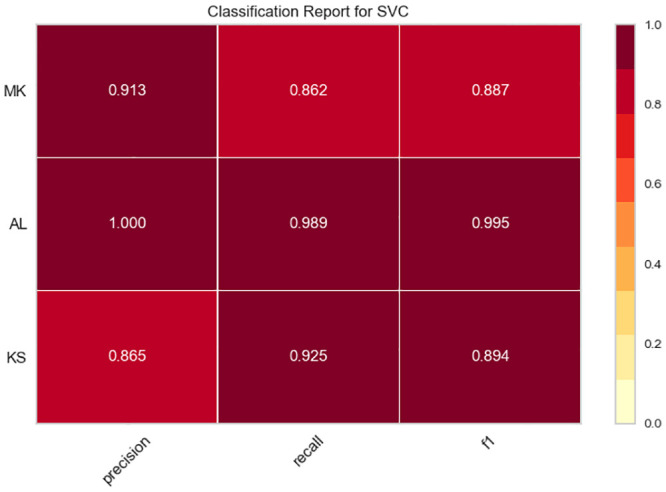
The classification reports for experiments with Twitterer-level models.

**Fig 7:**
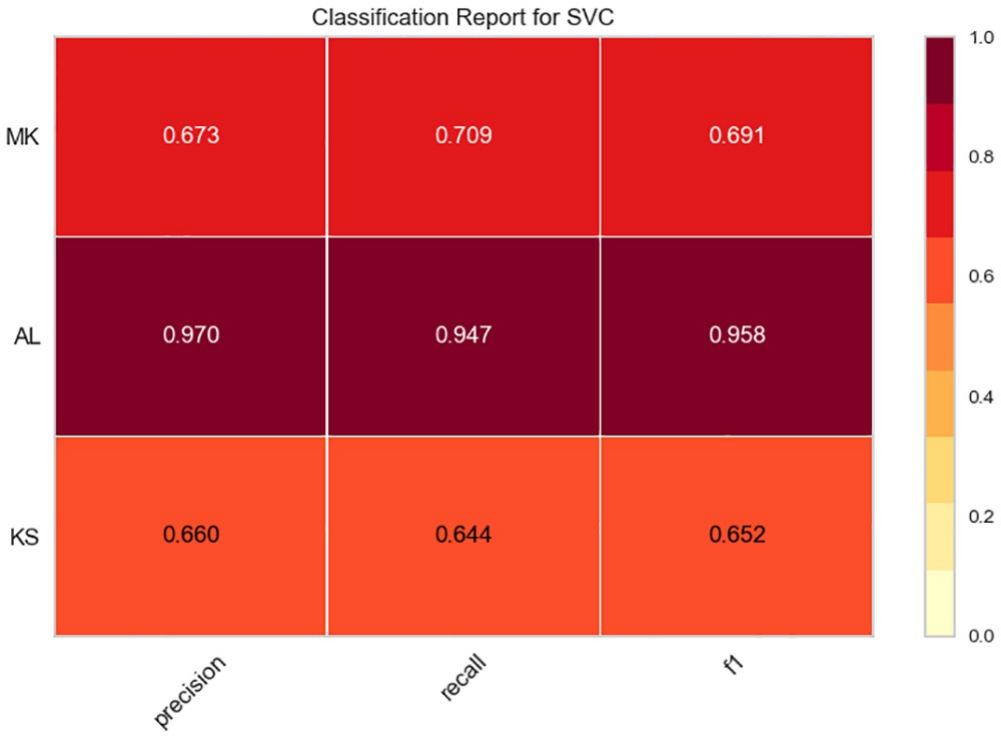
The classification reports for experiments with tweet-level classification models.

The classification report visualizer displays the precision, recall, F1, and support scores for the model. Classification reports for experiments with Twitterer-level and tweet-level classification models are shown in Figs 6 and 7. To support easier interpretation and problem detection, the report integrates numerical scores with a color-coded heat map. All heat maps are in the range (0.0, 1.0) to facilitate the comparison of classification models across different classification reports.

### 5.3 The learning curve

In this subsection, we present the learning curves as the relationship between the training score and the cross-validated test score for the SVC estimator with varying training samples.

**Fig 8:**
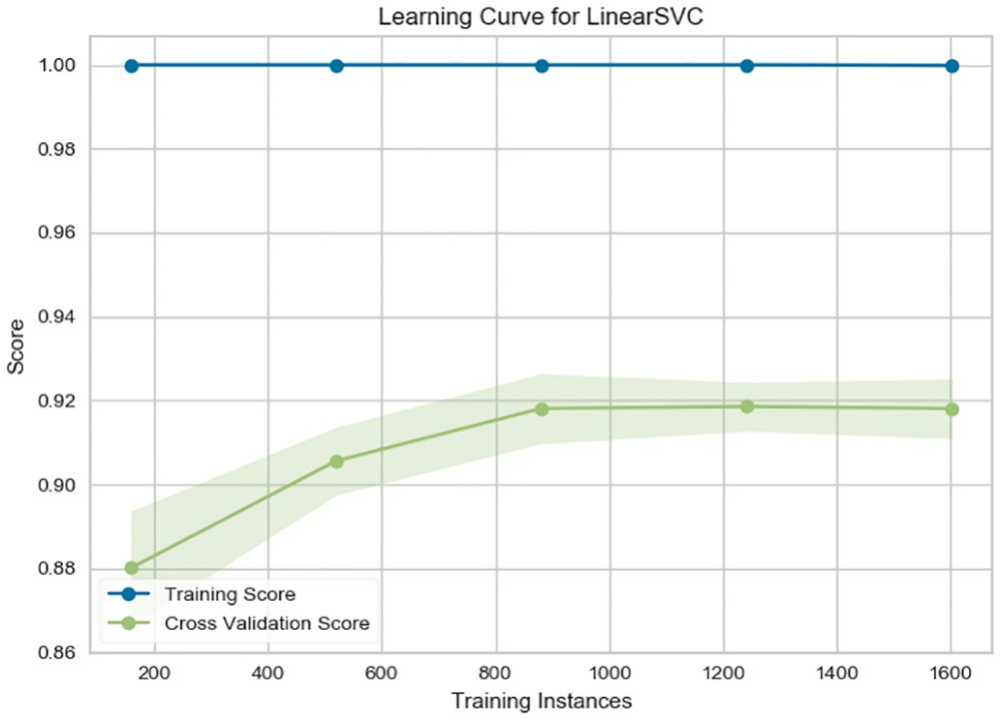
The learning curve for SVC trained on Twitterer-level TF-IDF modeling.

**Fig 9:**
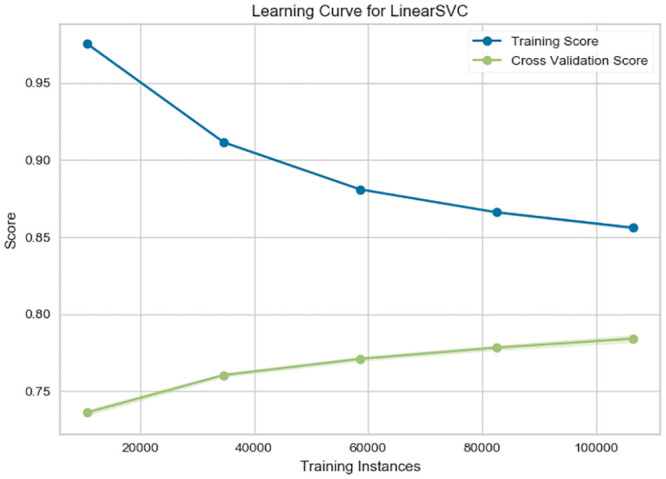
The learning curve for SVC trained on individual tweet-level TF-IDF modeling.

Since the training score in Fig 8 is much greater than the validation score, the model probably requires more training examples to generalize more effectively. On the other side, Fig 9 shows a proper convergence.

### 5.4 The class prediction error analysis

**Fig 10:**
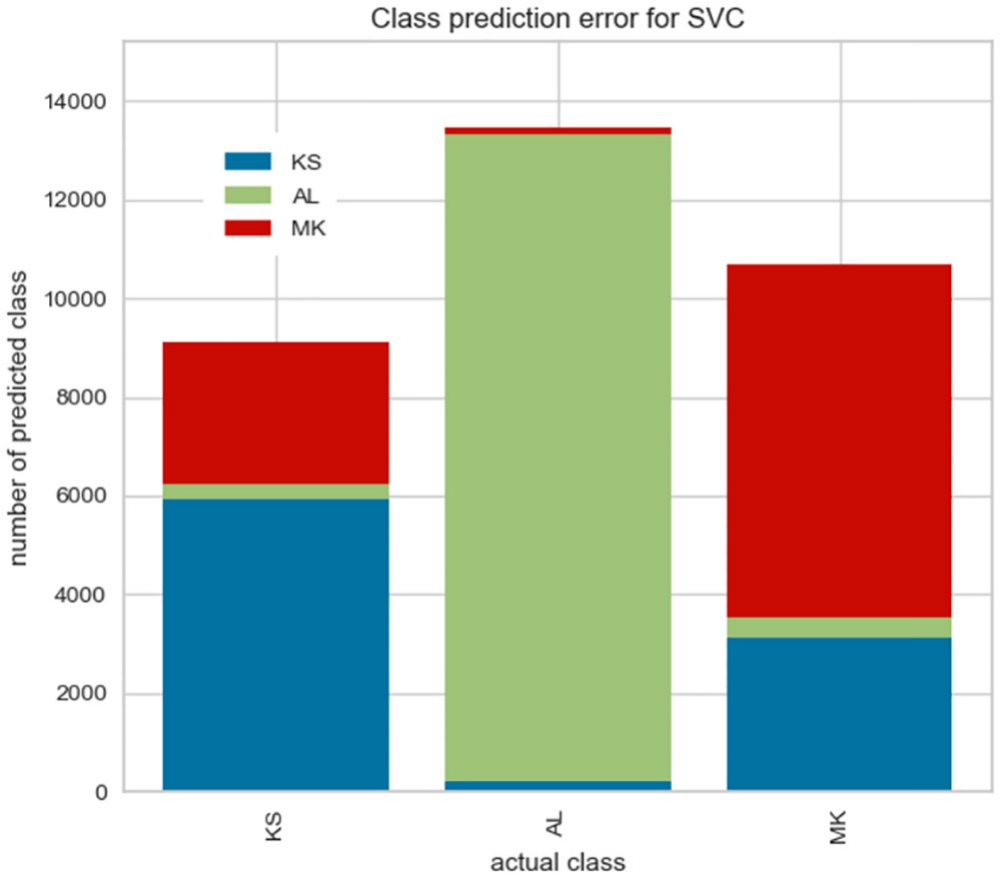
The class prediction error analysis for Tweeterer-level model.

**Fig 11:**
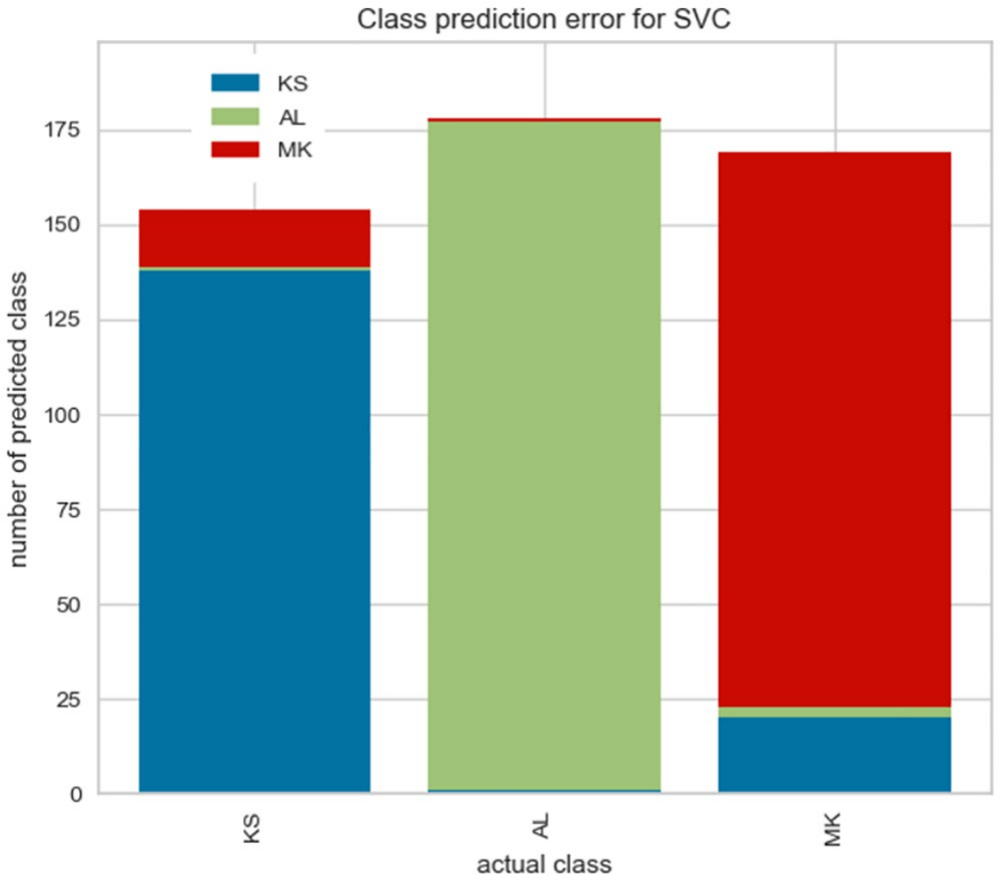
The class prediction error analysis for Tweet-level models.

We take a closer look at our best-performing classifier by plotting class prediction error plots in Figs 10 and 11. This plot shows the number of training samples (support) for each class in the fitted classification model as a stacked bar chart. Each bar is segmented to show the proportion of predictions (including false negatives and false positives, like a Confusion Matrix) for each class.

We observe that the two most problematic dialects–Gheg dialects used in regions of Kosovo (KS) and North Macedonia (MK)–are being confused with each other.

### 5.5 Archetypal feature analysis

Next, we inspect the top 25 significant features. We used archetypal analysis (AA) on the TF-IDF matrix to identify the top archetypal terms.

AA [60–62] is a technique used in unsupervised machine learning to identify the most representative examples, or archetypes, within a dataset. These archetypes can then be used to perform feature analysis by identifying the features that are most important for characterizing each archetype. Additionally, the archetypes themselves can be used as a basis for classification or regression models, allowing for a more intuitive and interpretable approach for machine learning.

**Fig 12:**
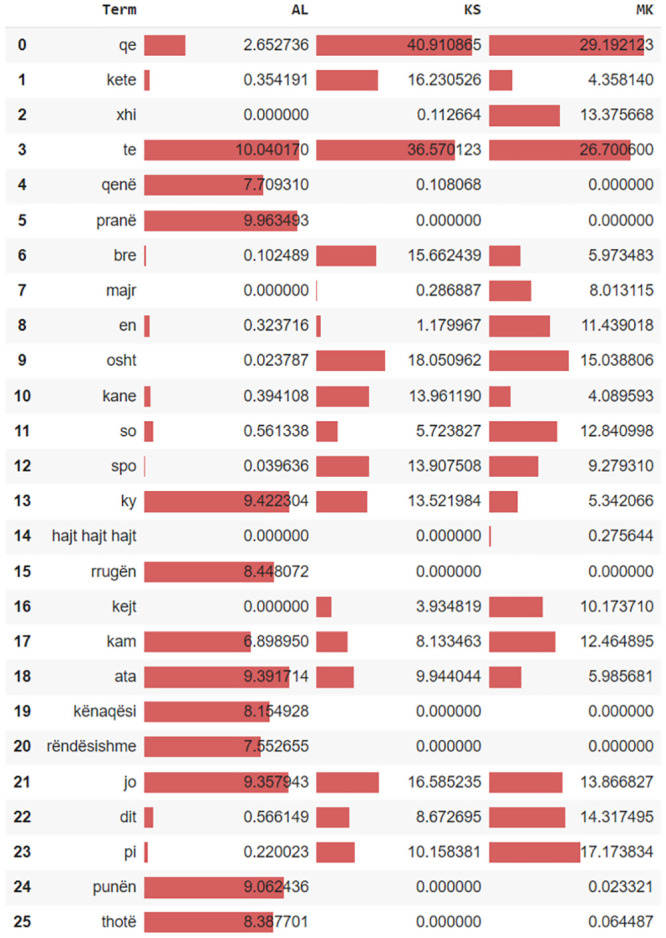
The most archetypal features.

Archetypal terms are those that are the most extreme linear combination of terms in the three-dimensional space representing the three Albanian dialects. For instance, in Fig 12 the terms “xhi” (row 2), “majr” (row 7), and ‘en’ (row 8) are mostly used in Gheg, especially in the region along the border of Kosovo and North Macedonia. Those terms are in a sense archetypal or extreme since they are best representatives of North Macedonian Gheg dialect terms. Similarly, the terms “qenë” (4) and “pranë” (5) are archetypally representing the Albanian Tosk dialect. In Fig 12 beside the term importance given by the row importance calculated via the AA communicates the relative a-posteriori parameter values for each language.

### 5.6 The human annotation results

We have asked 4 human subjects to manually annotate a subset of 330 randomly selected samples from the AlbDialectCorpus. All the subjects involved in the annotation task are native speakers of Albanian: two from Albania, one from Kosovo, and one from North Macedonia. All annotators understood the task and the presented examples in three dialects. The samples considered in the manual annotation process have been randomly selected, while aiming for a balanced distribution–for all three dialect identification tasks. Thus, a total of 330 samples have been selected, from which each of the regional dialects is represented by 110 instances.

Another fact about the dataset is that the samples considered for annotation are chosen in the following manner: in the case of tweet-level instances, the 330 examples are selected randomly from the whole set of 168,934 tweets. While for the user-level examples, we selected only 10 random samples for each user. This made the task more challenging from a human perspective, as we took away most of the context from the user-level annotation, with useful linguistic and semantic clues that could have provided great help in inferring the correct classes. However, at the same time, we aimed to reduce the annotation time by as much as possible, since all human subjects were volunteers providing the annotations for free. As we seek to fairly compare the human skills with the performance of the ML models in differentiating among dialects, we consider the results reported in the third evaluation scenario, in which the models are trained on sentences and tested on tweets. For the evaluation to happen in similar circumstances, the named entities in the samples presented to the human annotators have been replaced with the special token $ne$, just as in the data samples used to train and evaluate the ML models.

The summary of the human annotation is presented in Table 5. For dialect identification on both types of data, the worst results are way below the machine learning classifiers. We believe it is fair to compare the human performance at the user and tweet level with the performance of ML models applied to tweets.

**Table 5:** The accuracy rates and macro F1 scores of four human subjects that were asked to annotate 300 tweets and 30 users with dialectal labels. The last row indicates the average values computed on all four annotators.

Human Classified Data				
Annotator ID	Accuracy		macro F1-score	
	User-level	Tweet-level	User-level	Tweet-level
A1	0.73	0.70	0.70	0.67
A2	0.72	0.69	0.69	0.66
A3	0.75	0.72	0.71	0.69
A4	0.70	0.67	0.69	0.65
Average	0.73	0.69	0.70	0.66

**Fig 13:**
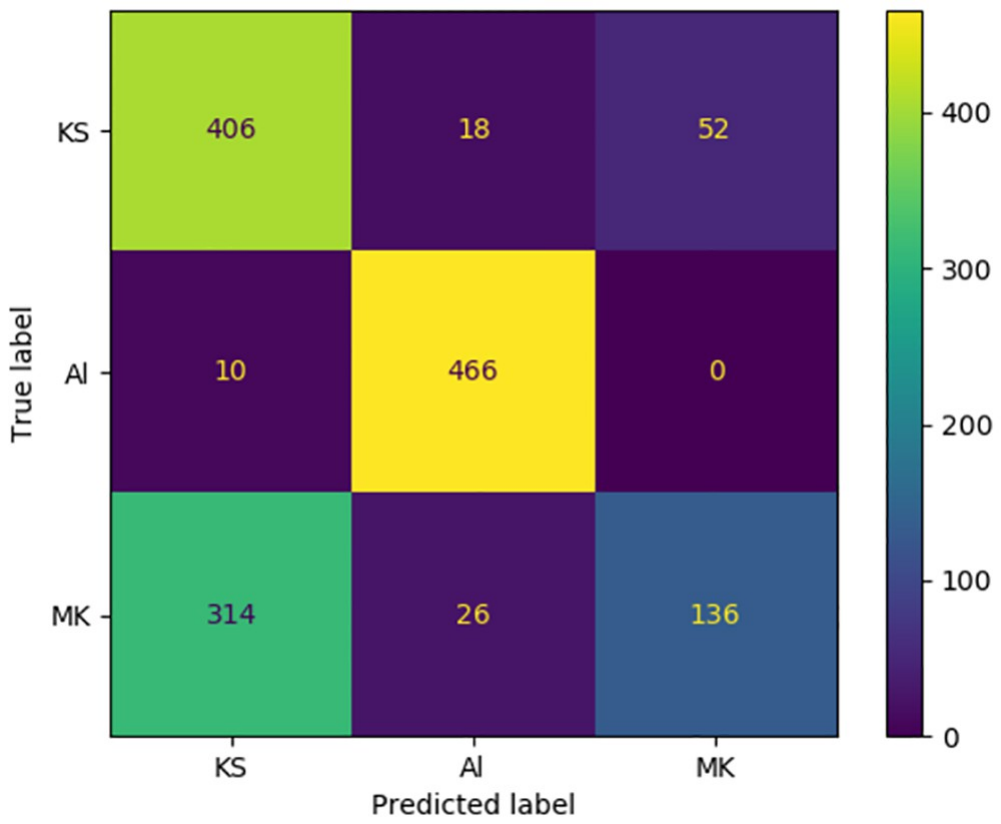
A sum of dialect identification confusion matrices computed on all annotators included in the human evaluation study.

Fig 13 displays the mean of confusion matrices computed for all annotators encompassed in our human evaluation study regarding the dialect identification task, irrespective of input data type. It is evident that annotators exhibit high confidence when presented with samples corresponding to the Albanian Tosk dialect. They demonstrated a markedly higher success rate in differentiating between Tosk/Standard Albanian and Gheg dialects. In a statistical context, 466 out of 476 samples are accurately classified, yielding a 0.98 accuracy rate.

Analogously, annotators exuded confidence when encountering samples associated with the Kosovo Gheg dialect. Quantitatively, 406 out of 476 samples are accurately classified, resulting in a 0.95 accuracy rate.

Conversely, when annotators were provided samples linked to one of the sub-Gheg dialects, their confidence levels diminished considerably. They frequently misclassified samples as Kosovo Gheg, despite their actual affiliation with the North Macedonian Gheg variant. This proclivity can be attributed to the inherent challenge in discerning these sub-dialects in the absence of explicit indicators, typically manifested as specific words or grammatical structures. Fig 13 reveals that a mere 136 out of 476 tweets are accurately classified, culminating in a 0.29 accuracy score. Furthermore, these suboptimal outcomes corroborate the arduous nature of this binary classification task from a human perspective.

In Table 6, we exhibit six samples selected from the dataset provided to human annotators. The first two samples pertain to the Tosk dialect, the subsequent two represent the Gheg (Kosovo) sub-dialect, and the final two samples correspond to the Gheg spoken in North Macedonia.

**Table 6:** Sentences that have been labeled with ground-truth labels for the dialect identification task, along with the labels assigned by humans, are provided along with their corresponding English translations for improved understanding.

Tweet ID	Ground Truth	Labels by Annotators			
		A1	A2	A3	A4
T1	AL	AL	AL	AL	AL
T2	AL	AL	AL	AL	AL
T3	KS	KS	KS	KS	KS
T4	KS	KS	MK	MK	MK
T5	MK	MK	MK	MK	MK
T6	MK	KS	KS	KS	MK
**Tweet**	**Original Tweet**	**English Translation**			
T1	Mësoni fakte rreth koronavirusit të ri	Learn facts about the new coronavirus			
T2	Mëngjes gushti në Parkun e Madh!	August morning in the Great Park!			
T3	Dil e voto. Percakto vet fatin tond per 4 vitet e ardhshme!	Go out and vote. Determine your destiny for the next 4 years!			
T4	Po pritojj bre ajde mloma	I’m getting lazy, cover me up			
T5	Xhi nuk mba kjo $ne$ e shkret	What this desolate *ne* does not hold			
T6	mos, se hazer jam	Do not, since I am ready			

Additionally, Table 6 encompasses English translations of each sample to facilitate comprehension. The samples were chosen based on two distinct scenarios: (c1) when the majority of annotators concur on a label that aligns with the ground-truth label; (c2) And when the majority of annotators concur on a label that diverges from the ground-truth label.

Tweets T1, T2, T3, and T5 exemplify case (c1), as the majority of votes correspond to the accurate label. These samples contain explicit cues indicating their association with the Moldavian dialect. For T1 and T2, in addition to vocabulary clues, there is evident syntactic and semantic accuracy, a primary characteristic of the Standard Tosk dialect. In T3, the words are typical of the Kosovo Gheg dialect. Lastly, T5 encompasses the words ‘xhi’ and ‘mba’, which serve as robust indicators for the North Macedonian Gheg dialect. For these and other term signals, refer to Fig 12.

Tweets T4 and T6 typify case (c2). Both samples lack linguistic or semantic clues to suggest that the sentences belong to the specific Gheg dialect.

### 5.7 Individual feature analysis

The SHapley Additive exPlanations (SHAP) methodology offers a means to quantify the influence of distinct input variables on a machine learning model’s output. This approach facilitates the interpretation of model predictions by policymakers and other stakeholders, allowing them to discern the contributing factors in a prediction.

SHAP supplies an array of valuable instruments for interpreting machine learning models, such as waterfall plots and summary plots. These tools enable visualization of each feature’s contribution to the model’s prediction, which is particularly beneficial for stakeholders lacking a machine learning background who seek insight into the driving forces behind the model’s predictions.

Two observations were isolated and the SHAP values were calculated for them, as displayed in Table 7. Representatives from Kosovo and North Macedonia were selected, while those from the Albanian (Tosk) dialect were excluded due to previous experiments demonstrating that classification models exhibit high confidence when determining this category.

**Table 7:** Two representative tweet sets extracted from the corpus: Each corresponding to the Twitterer from KS and the MK dialects/regions.

Region	Users’ Tweets from a given Region
Kosova	zani saj mban mu ni ma mir se qysh jam per momentin ndarja jon osht gabimi ma i madh qe mujm me ba Perhajr bajrami edhe juve ju koft perhajr Qe qetu tu hanger bakllav tinxa kurdo qe tmungoj qoma adresen kqyrni ku po bini ndashni se me dal nuk o leht Veq pi zorit tpata sha Nashta neser hale mgjen ama sot sjam tut mungu
North Macedonia	Tag inspektorin xhi e njofish. Xhi o puna e Ksaj festë Birra Korça, di kapim pushkat? Se dikujt ja kan chu nboten qeter denjo tfamiljes tu e ngj. Tag folirontin xhi ka full folija nrab!! Njerzit ma tkçi n’planet jonçato xhi ene tçojn Snap po eneçat xhi taçon e vnon nstory. N’Kulem t’Veres ma frajk e xhon njeri 1 nause se sa 1 kamarier. Tha njoni xhi kisht restaurant. Taj nuk ije orospaje Taj ije zemer majr Nauk se kije qif tdalish me sicallin gjal ama tvajn gjinah tva prajshish.

**Fig 14:**
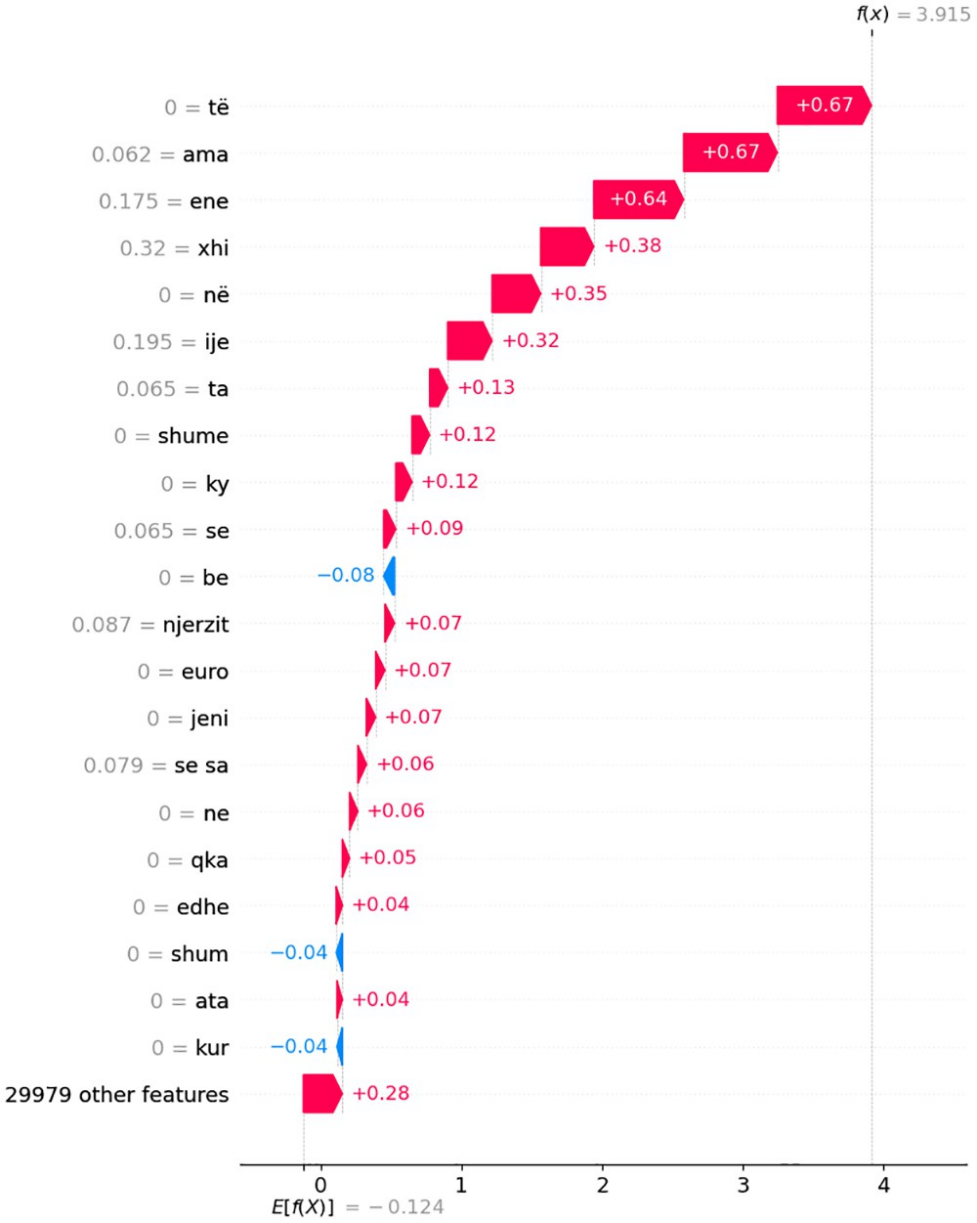
Depiction of waterfall SHAP visualizations for a pair of exemplary observations derived from Table 7 in a comparative manner for MK dialect.

**Fig 15:**
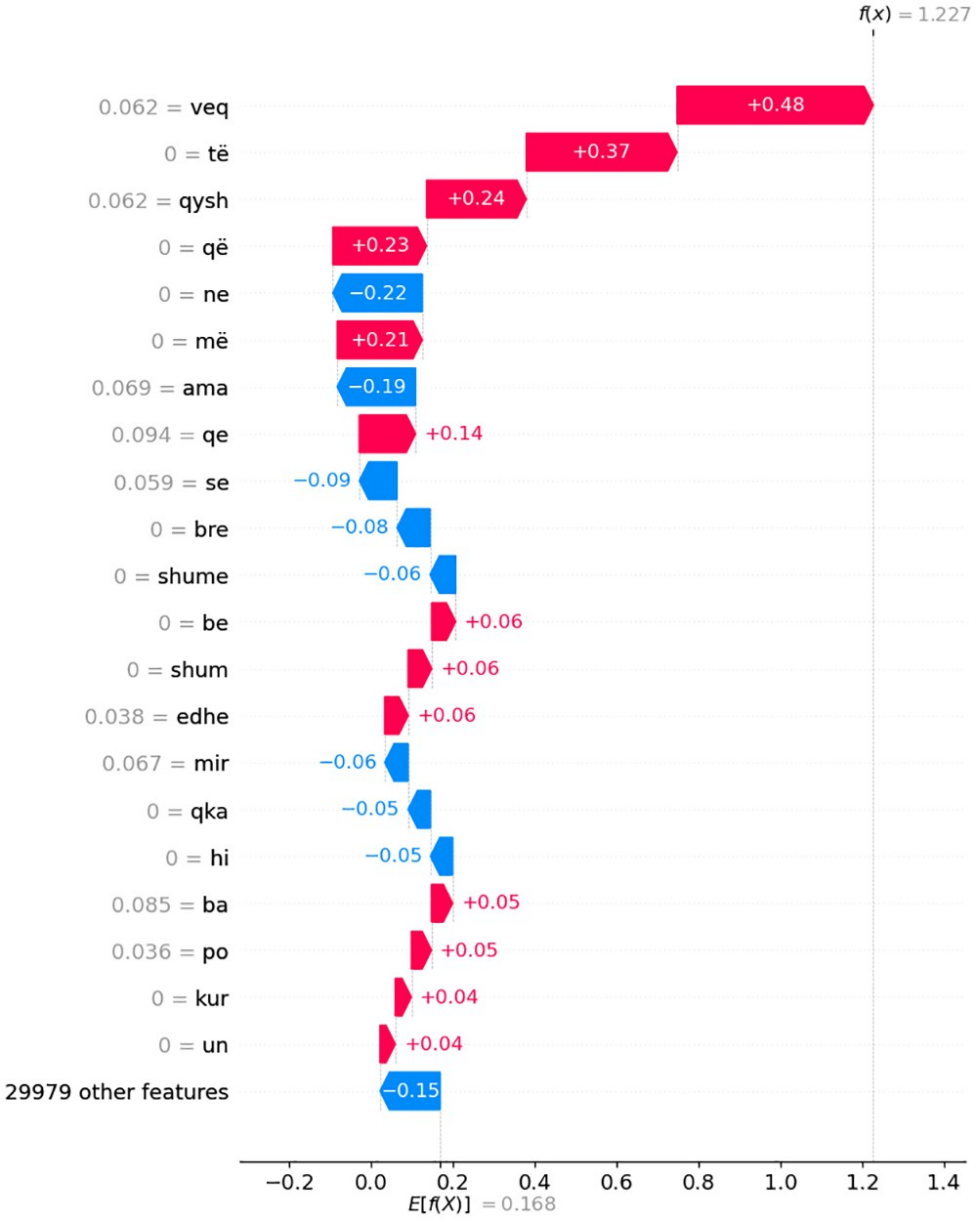
Depiction of waterfall SHAP visualizations for a pair of exemplary observations derived from Table 7 in a comparative manner for KS dialect.

**Fig 16:**
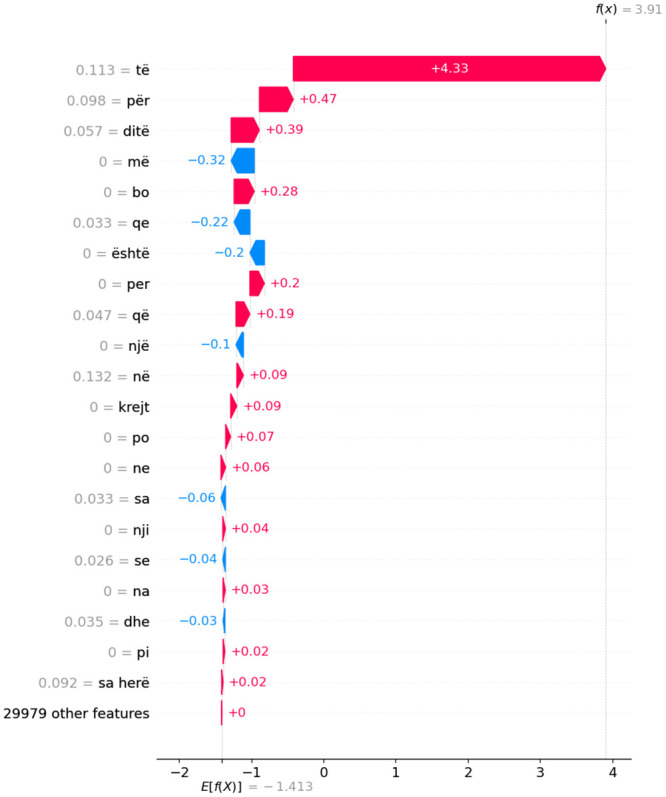
Depiction of waterfall SHAP visualizations for a pair of exemplary observations derived from Table 7 in a comparative manner for AL dialect.

Figs 14–16 present waterfall SHAP visualizations for the two representative observations from Table 7. The x-axis represents the target (dependent) variable values, which correspond to the dialect class. In this context, *x* signifies the chosen observation, *f*(*x*) denotes the model’s predicted value given input *x*, and *E*[*f*(*x*)] signifies the target variable’s expected value.

Absolute SHAP values indicate the extent to which an individual feature influenced the prediction. For instance, in the first subfigure a) of Fig 14, features **ama**, **ene**, and **xhi** contributed most significantly to the prediction of the MK dialect. The input variables’ impact on individual observations for dialect classification model output corroborates the findings presented in subsection 5.5. A meticulous reader can verify the recurrence of the most salient individual-level features delineated in this chapter, as well as in Fig 12 featured in subsection 5.5.

## 6 Conclusion

Twitter can be used to collect dialectal tweets for each Albanian-speaking country with high accuracy for some countries and average accuracy for other countries.

This paper presents a novel and comprehensive multi-dialectal Albanian corpus, which has been compiled using an automated scrapping methodology, capturing user data from three regions: Albania, Kosovo, and North Macedonia. The dataset–which has been anonymized for public use–consists of user data, additional user data, users’ tweets, tweets-related auxiliary data, and annotations aligned to dialect classes. Our work has not only provided a valuable resource for the study of Albanian dialects but also demonstrates the feasibility of using advanced geotagging techniques to facilitate corpus creation.

Through extensive experimentation with various classification methods, feature engineering, and feature selection, we have showcased the efficacy of machine learning algorithms in discerning Albanian dialects at the granularity of individual tweets. In-depth analysis of the highest-performing algorithms has offered valuable insights into their decision-making processes–revealing numerous dialectal patterns that, while known to human annotators, have been largely overlooked within the broader research community. This study, therefore, not only advances our understanding of Albanian dialectal variation it also highlights the potential of machine learning in the analysis and interpretation of complex linguistic phenomena.

In the proposed study, several limitations warrant further discussion. One primary concern is the accuracy of advanced geotagging techniques, which may produce false positives or negatives, particularly for users with networks that span multiple regions. Additionally, the sample size, although substantial, may not adequately represent the full range of dialectal variations within the Albanian-speaking community. Furthermore, the inherently brief and context-limited nature of tweets complicates dialect identification, posing a challenge to both human and machine classifiers. Temporal variability also emerges as a concern; the Albanian language and its dialects evolve over time, which means the current dataset could quickly become outdated.

External validity remains a limitation as the method’s effectiveness in classifying dialects may not generalize to other forms of written or spoken Albanian. Moreover, the data collection, annotation, and analysis processes could be resource-intensive, which might be a constraint for researchers with limited resources. Lastly, the use of human annotators as a benchmark for machine learning performance could skew results. If these annotators lack expertise in Albanian dialects, their lower accuracy rates could artificially inflate the perceived effectiveness of machine learning models. These limitations suggest avenues for further research and improvements in the methodology.

Despite the achievements of this study, several areas warrant further investigation and improvement. For future work, we propose the following two research directions: 1) To increase the robustness of the classification models, we aim to expand the dataset by incorporating more dialects and user data from additional regions where Albanian is spoken, which will facilitate a deeper understanding of the linguistic diversity within the Albanian language; and 2) To collect more data from a broader range of sources, such as social media platforms other than Twitter, and incorporating written and spoken texts–which would enable a more comprehensive understanding of the Albanian dialect landscape. This expansion would also contribute to the generalizability of the developed classification models.
